# Help or harm? Assessing positive and unwanted effects of a self-guided internet-based intervention for gambling problems^☆^

**DOI:** 10.1016/j.invent.2025.100893

**Published:** 2025-12-02

**Authors:** Lara Rolvien, Duygu Sönmez, Lea Schuurmans, Lisa Borgmann, Annika Schmüser, Steffen Moritz, Anna Baumeister

**Affiliations:** a Department of Psychiatry and Psychotherapy, University Medical Center Hamburg-Eppendorf, Martinistrasse 52, 20246, Hamburg, Germany

## Abstract

**Background:**

Self-guided internet-based interventions have been established as effective tools for reducing gambling disorder. However, research on their potential negative side effects remains limited. This study investigated both unintended positive effects, and unwanted negative effects, of the self-guided internet-based intervention ‘Restart’ for individuals with self-reported gambling problems.

**Methods:**

A total of 94 participants with self-reported gambling problems were investigated for positive and unwanted effects using the Positive and Negative Effects of Psychotherapy Scale for Internet-Based Intervention (PANEPS-I) after using the internet-based intervention ‘Restart’ for six weeks.

**Results:**

The findings indicate that positive effects, such as increased self-efficacy (64.8 %), were reported more frequently than unwanted effects. Nonetheless, a significant proportion of participants experienced side effects (23.9 %), alleged malpractice (38.6 %), and found procedures unethical (21.5 %). Key unwanted effects included feelings of stigmatization and blaming, experiences of excessive pressure during the intervention and perceptions of the program as overly directive or as not in line with own preferences. Positive effects but not unwanted effects significantly increased the likelihood for a positive response (Odds ratio = 1.59 *p* < .001).

**Conclusion:**

The findings suggest that while self-guided internet-based interventions can serve as valuable tools for individuals reluctant to seek traditional therapy, they require careful refinement to address user-specific needs and reduce adverse outcomes. Future research should focus on identifying factors that predispose individuals to positive and unwanted effects and explore intervention modifications, such as integrating optional therapeutic support or personalized content adaptations.

## Highlights

• The study evaluated unintended positive and negative effects of a self-guided online intervention for gambling problems.
• 83.2 % of participants reported at least one positive effects (e.g., increased self-efficacy).
• Notable negative effects included feelings of stigmatization, excessive pressure, and perceptions of unethical procedures.
• Positive unintended effects significantly predicted intervention success; negative unintended effects did not.
• The findings emphasize the need for refining digital interventions to enhance benefits and reduce harm.

## 1 Introduction

Self-guided internet-based interventions have been shown to be effective in reducing symptoms of gambling disorder with moderate effect sizes (Hedges' *g* = 0.73; Sagoe et al., 2022). However, negative or unwanted effects have hardly been investigated for this disorder and type of intervention, as most studies to date have focused primarily on their effectiveness in reducing symptoms of gambling disorder. Despite the well-established advantages of internet-based interventions (Andersson et al., 2019; Andersson and Cuijpers, 2008; Andersson and Titov, 2014), unwanted effects can also occur (Boettcher et al., 2014; Karyotaki et al., 2018; Rozental et al., 2015). Potential unwanted effects observed during internet-based interventions must, at minimum, be equated with those encountered in traditional face-to-face interventions (Andersson et al., 2019). Hoffmann et al. (2008) thoroughly outlined potential unwanted effects in psychotherapy, which include deterioration or chronification of targeted symptoms, emergence of new symptoms, suicidality, and decreased self-esteem and self-efficacy due to unmet (possibly unrealistic) treatment aims, among others. As part of a questionnaire developed to assess unwanted effects and unintended positive effects of psychotherapy, Peth et al. (2018) identified a four-factor structure comprising ‘positive effects’, ‘side effects’, ‘malpractice’, and ‘unethical procedure’ (see Table 1), which are examined in the present study.

**Table 1:** Conceptualization and classification of unintended positive and unwanted effects in psychotherapy (defined by Peth et al., 2018).

Categories	Description
Positive effects (PE)	Positive developments such as changes in self-confidence, personal responsibility, performance, emotional resilience, readiness for further psychotherapy, and pride in personal achievements
Side effects (SE)	Negative consequences that emerge during correctly applied treatment, including concerns about stigma or alterations in one's relationships with family and friends
Malpractice (MP)	Incorrectly applied treatment, including improper application of therapeutic techniques or the acquisition of problematic behaviors
Unethical procedure (UP)	Inappropriate actions or use of language of the therapist, including an intolerance toward aspects such as gender, religion, or sexual orientation

### 1.1 Unwanted effects of internet-based psychotherapeutic interventions

Just like any potentially effective treatment, internet-based Cognitive Behavioral Therapy (iCBT) may also yield undesirable outcomes or fail to halt ongoing deterioration (Karyotaki et al., 2018). Boettcher et al. (2014) unveiled a range of unwanted effects in internet-based interventions, spanning from emergence of new symptoms and deterioration of targeted symptoms to negative well-being and lack of apparent treatment results. These can lead to potential non-compliance, premature termination of treatment, alterations in work situations, and fears of stigmatization (Boettcher et al., 2014). These unwanted effects, though studied in the context of social anxiety disorder, could potentially manifest across various disorders. The majority of self-help interventions cannot be adjusted to the individual's characteristics (e.g., education level, symptom severity, and personal preferences) or possibly changing conditions (e.g., symptom deterioration). Moreover, difficulties with the implementation of the tasks and perceived lack of therapeutic support might result in user frustration, which, in turn, can promote adverse effects related to the program (Fenski et al., 2021). According to three meta-analyses, iCBT interventions yielded symptom deterioration in 3.6 % to 5.8 % in cases of depression (Ebert et al., 2016; Karyotaki et al., 2018); similar observations have been made in other disorders (Rozental et al., 2017; Ebert et al., 2016; Karyotaki et al., 2018). For example, in a study investigating a 6-week internet-based intervention targeting mild to moderate depression, Fenski et al. (2021) found that 8.6 % of the participants reported unwanted effects. In line with field-level recommendations (Rozental et al., 2018), we define unwanted effects as adverse experiences that are distinct from treatment errors or deterioration. Rozental et al. (2018) emphasize that systematic monitoring throughout treatment is ideal to detect such effects, but in the present study we focus on participants' post-intervention experiences only.

### 1.2 Unwanted effects of internet-based interventions for gambling problems

Although there are some research efforts to investigate unwanted effects of internet-based interventions for several mental illnesses, such as depression and anxiety disorders, empirical evidence is lacking for gambling disorder. Delving into gambling disorder, it bears similarities with substance-related addictions, a view reinforced by the diagnostic reclassification in the ICD-11, which shifted it from the impulse-control disorders to the behavioral addiction disorders section (World Health Organization, 2018). Wins, regardless of magnitude, activate the reward system, initiating a cascade of hormones, including dopamine and serotonin (Potenza et al., 2019). Therapeutic interventions, such as advocating abstinence, can induce withdrawal symptoms like inner restlessness and dissatisfaction, potentially intensifying cravings for additional gambling experiences (Fernandez et al., 2020), which should be carefully considered. Furthermore, the treatment of multimorbid disorders is particularly complex, as gambling disorder often occurs together with other substance use disorders, depression or anxiety disorders (Grant and Chamberlain, 2020). In addition, a low level of education and limited insight into the disease or awareness of the problem may augment rates of unwanted effects.

Consequently, standardized, self-guided formats may not adequately address the multifaceted aspects of an individual's condition, potentially resulting in the aforementioned unwanted effects of negative well-being and non-compliance with treatment. Thus, while self-guided internet-based interventions offer a scalable and low-threshold solution to gambling issues, a comprehensive understanding of their impact, embracing both positive and unwanted effects, is indispensable.

### 1.3 The current study

The current study aims to exploratory investigate the unintended positive and unwanted effects of the internet-based intervention ‘Restart’ for individuals with self-reported gambling problems. In addition, the potential influence of positive and unwanted effects on responding to the intervention will be examined. The data were collected within a larger randomized controlled trial (RCT, German Clinical Trials Register registration number DRKS00024840) to test the effectiveness and acceptance of the intervention (Rolvien et al., 2024).

## 2 Methods

### 2.1 Study design

The initial study was set up as an RCT with two online assessments (baseline and 6 weeks after baseline) and two treatment arms (intervention and a wait-list control group). Within an intervention period of six weeks, the self-guided internet-based intervention ‘Restart’ for individuals with gambling problems could be used. For a detailed description of the study setup and the measurement instruments at each time point see (Rolvien et al., 2024). The current study focuses on the characteristics and gambling symptoms of the intervention group at baseline and post-intervention (see Section 2.3 ‘Instruments’), as well as the assessment of positive and unwanted effects six weeks after the baseline assessment among completers.

### 2.2 Participants

Participants were recruited through various social media platforms such as Facebook and Instagram, as well as by distributing small flyers in gambling establishments across Germany. The first participant enrolled on July 13, 2021, and the last participant completed the post assessment on March 6, 2023. To be eligible for participation, individuals had to meet the following criteria: (a) self-reported need for assistance due to gambling-related issues, (b) access to the internet, (c) aged between 18 and 75 years, (d) a reasonable command of the German language, (e) willingness to engage in two anonymous online evaluations, and (f) the provision of informed consent. Exclusion criteria encompassed a lifetime diagnosis of schizophrenia, bipolar disorder, and the presence of acute suicidal symptoms (assessed using the final item of the PHQ-9 with a score > 2). In cases of acute suicidality, support hotlines and resources were made available.

### 2.3 Instruments

#### 2.3.1 Pathological Gambling Adaptation of the Yale-Brown Obsessive-Compulsive Scale

The Pathological Gambling Adaptation of the Yale-Brown Obsessive-Compulsive Scale (PG-YBOCS; Pallanti et al., 2005) was used to assess gambling disorder symptom severity and served as primary outcome measure in the RCT. The questionnaire comprises two distinct subscales, each consisting of 5 items designed to determine the extent of gambling severity over the past week. The first subscale is centered around the assessment of thoughts and urges related to gambling, while the second subscale focuses on evaluating actual gambling-related behavior. The total score on the PG-YBOCS can range from 0 to 40, with scores in the range of 0–7 indicating subclinical severity, scores between 8 and 15 signify mild severity, scores between 16 and 23 indicate moderate severity, scores between 24 and 31 reflect severe severity and scores within the range of 32 to 40 denote extreme symptom severity. The PG-YBOCS exhibits a high internal consistency of Cronbach's α = 0.97 (Pallanti et al., 2005).

#### 2.3.2 Positive and Negative Effects of Psychotherapy Scale for Internet-based Interventions (PANEPS-I)

The Positive and Negative Effects of Psychotherapy Scale for Internet-based Interventions (PANEPS-I; Baumeister et al., 2024) is a modified iteration of the Positive and Negative Effects of Psychotherapy Scale (PANEPS; Peth et al., 2018). The PANEPS-I serves as a self-assessment questionnaire utilized to assess both positive and unwanted effects of psychotherapeutic internet-based interventions covering four dimensions, namely positive effects (PE), side effects (SE), malpractice (MP), and unethical procedure (UP). The items are answered on a 4-point Likert scale, ranging from 1 (agree) to 4 (disagree), as well as an additional response category of 5 (not applicable). An overall mean score can be computed for all dimensions except for PE (due to its distinct polarity). Additionally, the frequency of agreement per item can be evaluated by dichotomizing responses (1 and 2 = agreement, 3 and 4 = disagreement). Agreement on the PE scale signifies the presence of beneficial outcomes resulting from the intervention, while agreement on the other scales indicates the presence of aversive effects arising from the intervention. The SE, MP, and UP subscales were adapted for use in a fully self-guided online program. Items refer to participants' subjective experiences of the program's content, tone, structure, or delivery, not to clinician behavior. SE capture perceived negative emotional or social consequences of program use (e.g., “The online program has worsened my relationship with my family/friends”). MP reflects perceived inappropriate or ineffective aspects of the program (e.g., “The online program made me feel like I was to blame for my problems”). Endorsement on the UP subscale reflects participants' perceptions of the program's ethical features, including privacy concerns, perceived pressure, and inclusivity of content (“I had concerns about the privacy of my data”; “I felt pressured by the online program to do things I didn't really want to do”; “The online program seemed intolerant of my origin, religion, sexual orientation, or gender”). The UP subscale captures subjective reactions to the program itself rather than clinician or research misconduct. “Not applicable” responses were treated as missing values (see Table 3 footnote) and excluded from mean score calculations. Cronbach's α coefficients in the present sample were 0.83 (total scale), 0.89 (PE), 0.82 (SE), 0.80 (MP) and 0.35 (UP). In regression analyses, for each subscale, a mean score was computed, with higher values indicating stronger agreement with negative effects, and a one-unit increase represents a one-point increase in the respective subscale mean score. The internal consistency of the PANEPS has been reported to range from Cronbach's α ranging from 0.72 to 0.92 (Moritz et al., 2019). The PANEPS-I demonstrated acceptable to good internal consistency for the subscales PE, SE, and MP, with Cronbach's α ranging from 0.70 to 0.85 (Baumeister et al., 2024).

### 2.4 Intervention

The ‘Restart’ internet-based self-help intervention comprises 13 modules designed to address problematic behaviors and cognitions associated with gambling issues, including impulse management and relapse prevention. In addition, it addresses emotional and psychosocial symptoms such as low self-esteem, sleep problems, reduced social competence, decreased activity levels, and depressive symptoms, thereby also covering relevant comorbidities (Bücker et al., 2019, Bücker et al., 2021; Rolvien et al., 2024). The program incorporates cognitive-behavioral therapy (CBT) strategies (Beck et al., 1979), mindfulness techniques (Khoury et al., 2013), and metacognitive elements (Gehlenborg et al., 2021; Jelinek et al., 2019). The modules offer text-based content enriched with video and audio clips, interactive exercises, and worksheets. Participants are encouraged to spend 30 to 60 min on each module, completing two modules weekly so that the total content can be completed in six weeks. The sequence in which they engage with the modules is at their discretion. ‘Restart’ operates as a self-guided internet-based intervention, allowing participants to contact a personal moderator for questions or technical assistance, without additional therapeutic support. The program logs participant login durations for monitoring. ‘Restart’ has been extensively enhanced based on qualitative feedback of the first RCT (Bücker et al., 2021) and was evaluated in its new, optimized version in a further RCT as effective in reducing gambling symptoms and depressive symptoms (Rolvien et al., 2024). Notable additions include a new module on sports betting and an initial motivation-building module utilizing motivational interviewing techniques (DiClemente et al., 2017; Yakovenko et al., 2015). At the start of the program, four fictional characters who are struggling with gambling problems are introduced and accompany users through the modules with sample stories and illustrations. Importantly, participants can now use the self-help smartphone app ‘COGITO’ alongside the browser-based online program. ‘COGITO’ offers short self-help exercises based on CBT, metacognitive training, and mindfulness, primarily designed to enhance overall mental well-being but with additional exercises tailored to gambling symptoms. For details on COGITO and its evidence in effectiveness of reducing depressive symptoms and increasing self-esteem see (Bruhns et al., 2021, Bruhns et al., 2023; Lüdtke et al., 2018; Moritz et al., 2024). The app's design and content have also been refined with new program packages and audio exercises. In this current study, the app was promoted in the initial email providing the login data for ‘Restart’ and prominently featured in the online program's main menu.

### 2.5 Statistical analyses

Statistical analyses were performed with IBM Statistics® 27. Descriptive analyses were used to describe the sample, and positive or unintended side effects. Responders were defined as participants with at least a 35 % reduction in their total score on the PG-YBOCS from baseline to post-assessment (Farris et al., 2013). To analyze the binary outcome data (responders [1] vs. non-responders [0] based on the presence of positive or unwanted effects) a logistic regression model was fitted to the data to examine the relationship between response and the four predictors PE, SE, MP and UP. The model was estimated using a binomial error distribution. Odds ratios (OR) were derived from the coefficients estimated by the model. They represent the multiplicative change in odds for a one-unit increase in a predictor. The logistic regression model included four predictors: number PE, SE, MP, and UP. Additional covariates such as baseline symptom severity, concurrent treatment, or program usage were not entered, as the available sample size was limited and the inclusion of several correlated predictors could have led to model overfitting. Model diagnostics included checks for multicollinearity (VIF), influential cases (Cook's distance), residual behavior (simulation-based tests and binned residuals), calibration (Hosmer-Lemeshow test, bootstrap calibration), and discrimination (AUC with 95 % CI). We additionally reported Tjur's R^2^ as an indicator of explanatory power and calculated average marginal effects (AMEs) to facilitate interpretation of the odds ratios. In addition, exploratory comparisons were conducted between responders and non-responders on key baseline variables (e.g., age, gender, baseline symptom severity) to examine whether treatment response was associated with initial characteristics. Between-group differences were tested using *t*-tests or χ^2^ tests, as appropriate.

## 3 Results

### 3.1 Sample characteristics

Sample characteristics are reported in Table 2. The final sample consisted of 119 participants of which 61.3 % (*n* = 73) were male and 88.2 % (*n =* 105) were German. The average age was 35.12 (*SD* = 10.86) years. 50.4 % (*n* = 60) of the participants reported having a school-leaving certificate. As for the treatment variables, seven participants (5.9 %) reported using psychotropic medication, nine participants (7.6 %) indicated their current usage of self-help, and 12 participants (10.1 %) reported that they are currently in psychotherapy. 27 participants (22.7 %) were diagnosed with a gambling disorder and nine participants (7.6 %) indicated currently being in gambling suspension. Psychopathology was assessed with the Pathological Gambling Adaptation of Yale-Brown Obsessive-Compulsive Scale (PG-YBOCS) with a mean total score of 16.77 (*SD =* 7.64), which represents a moderate gambling symptom severity.

**Table 2:** Baseline characteristics and psychopathology (*N* = 119). Frequencies and means, and standard deviations and percentages in brackets.

	*M* (*SD*); *N* (%)
Demographic characteristics	
Gender (% male)	73 (61.3)
Age in years	35.12 (10.86)
School leaving examination	60 (50.4)
Nationality (% German)	105 (88.2)
Professional status (% full-time employed)	58 (48.7)

Treatment variables	
Psychotropic medication	7 (5.9)
Current usage of self-help	9 (7.6)
Currently in psychotherapy	12 (10.1)
Diagnosed gambling disorder	27 (22.7)
Currently in gambling suspension	9 (7.6)

Psychopathology	
PG-YBOCS^a^ total score	16.77 (7.64)
PG-YBOCS behavior	8.18 (4.08)
PG-YBOCS thoughts	9.29 (4.25)

a Pathological Gambling Adaptation of Yale-Brown Obsessive-Compulsive Scale.

### 3.2 Unintended positive and unwanted effects

Positive and unwanted effects (PANEPS-I) were analyzed for 94 participants (78.9 %) of the intervention group who completed the post assessment. Frequencies of agreement on the four subscales PE, SE, MP and UP are shown in Table 3. Positive effects were reported considerably more often than unwanted effects. In total, 68 participants (83.2 %) reported at least one PE, and 49 (55.7 %) reported four to six PE. Across items, the most frequently endorsed negative experiences referred to perceived blaming tone (18.8 %), technical difficulties (14.5 %), feelings of embarrassment (13.3 %), excessive demands or performance pressure (13.3 %), and fear of having implemented exercises incorrectly (13.3 %). Concerns about data privacy (11.9 %) were also relatively common. These themes point to modifiable design aspects such as providing clearer guidance and error tolerance (to reduce fear of “doing it wrong”), ensuring a supportive rather than self-critical tone, adjusting pacing and task load to user capacity, and improving technical stability and data transparency.

**Table 3:** Unintended positive and unwanted effects assessed with the PANEPS-I for complete cases (*n* = 94). Percentages and frequencies of subjective agreement.

Positive effects	*n*	Agreement in % (*n*)
After completing the online program, I gained confidence in my own abilities.	88	64.8 (57)
I learned to take responsibility for myself during the online program.	85	63.5 (54)
My performance has improved as a result of the online program.	85	57.7 (49)
I am proud of myself for completing the online program.	84	60.7 (51)
Through the online program, I was able to end stressful relationships.	85	47.1 (40)
Through the online program, my readiness for direct psychotherapy with a therapist has increased.	85	45.9 (39)

Side effects		
I am afraid that my social network will find out that I have been doing an online program.	83	6.0 (5)
I am embarrassed that I have done an online program.	83	13.3 (11)
The online program has worsened my relationship with my family/friends.	84	7.1 (6)
It bothers me that my family/friends treat me differently since I did the online program.	82	9.8 (8)
Since using the online program, I feel labeled as a mentally ill person.	84	4.8 (4)
My family/friends are embarrassed that I did an online program.	82	7.3 (6)
I'm afraid that I didn't correctly implement certain exercises or understand the content and that, as a result, my condition worsened.	83	13.3 (11)

Malpractice		
The online program only worked toward “eliminating the problem”; there was no positive goal orientation.	83	10.8 (9)
The online program did not (sufficiently) address my personal problems.	82	7.3 (6)
The online program put too much time and/or performance pressure on me.	83	13.3 (11)
I experienced technical difficulties with the online program that bothered me.	83	14.5 (12)
I did not feel addressed by the online program (e.g., due to lack of gender-appropriate language or lack of individuality).	80	8.8 (7)
I felt that my problems were not taken seriously in the online program.	84	7.1 (6)
The online program made me feel like I was to blame for my problems.	80	18.8 (15)
I learned behaviors through the online program that were very harmful to me (e.g., saying no excessively, setting myself apart).	82	12.2 (10)
From my point of view, the applied therapeutic techniques of the online program were wrong.	82	9.8 (8)
I feel that I have changed negatively as a result of the online program.	83	4.8 (4)
The exercises and information in the online program did not match what my therapist or doctor told me.	74	13.5 (10)

Unethical procedure		
I had concerns about the privacy of my data.	84	11.9 (10)
I felt pressured by the online program to do things I didn't really want to do.	83	12.1 (10)
The online program seemed intolerant of my origin, religion, sexual orientation, or gender.	82	6.1 (5)

*Note.* Missing values represent the choice of the response option “not applicable”. Agreement in percentages are valid percentages.

At least one SE was reported from 21 participants (23.9 %), with ten participants (11.4 %) reporting one SE, four (4.5 %) reporting two SE, three (3.4 %) reporting three SE, two (2.3 %) reporting five SE and both one (1.1 %) participant reporting six and seven SE. In sum, 34 participants (38.6 %) reported at least one MP, with 14 (15.9 %) reporting one MP, 11 (12.5 %) two MP, two (2.3 %) three MP, five (5.7 %) four MP, four (4.5 %) six MP and one participant (1.1 %) reported ten MP. In total, 19 participants (21.5 %) reported that at least one UP occurred, while 14 (15.9 %) reported one UP and five (5.7 %) two UP.

### 3.3 Responders versus non-responders

In total 46 (48.9 %) participants were identified as responders with at least 35 % symptom reduction on the PG-YBOCS at the post assessment. Logistic regression revealed that only PE significantly increased the odds of being a responder (OR = 1.59, *p* < .001). This indicates that for each additional unit of PE, the odds of being a responder increase by approximately 58.6 %, holding all other predictors constant. The other predictors SE (OR = 0.856, *p* = .563), MP (OR = 0.922, *p* = .723), and UP (OR = 0.677, *p* = .584) did not show statistically significant associations with the outcome. The model demonstrated good explanatory power, as indicated by Tjur's *R*^2^ = 0.31, which reflects a substantial ability to discriminate between responders and non-responders. Multicollinearity was low (VIF range 1.00–2.55), and no influential observations were identified. The simulation-based residual test suggested no major model misfit (*p* = .54). A mild deviation was indicated by the binned residual plot, which is not uncommon in small samples. Targeted linearity checks (Box-Tidwell terms) did not reveal significant departures from linearity in the logit. Calibration was acceptable (Hosmer-Lemeshow χ^2^(6) = 11.84, *p* = .07) and bootstrap calibration showed a small mean absolute error of 0.07. The model demonstrated good discrimination (AUC = 0.80, 95 % CI 0.70–0.90). The AME for PE was 0.079 (SE = 0.011, *p* < .001), meaning that each additional positive side effect was associated, on average, with a 7.9-percentage-point higher probability of being a responder, holding other predictors constant. AMEs for SE, MP, and UP were small and not statistically significant. To further illustrate the effect of PE, predicted response probabilities were computed across the observed PE range. The probability of being a responder increased steadily from 21 % (95 % CI = 10–38 %) at PE = 0 to 80 % (95 % CI = 64–90 %) at PE = 6.

### 3.4 Exploratory comparisons between completers and non-completers

Exploratory analyses were conducted to examine whether study completion was associated with baseline participant characteristics. Completers and non-completers did not differ significantly in sociodemographic variables and baseline symptom severity etc. (all *p*s > 0.05). A significant difference emerged only for the variable “currently in gambling suspension,” with a higher proportion of non-completers reporting an active gambling suspension at baseline, χ^2^(2) = 23.25, *p* < .001. Detailed results of these comparisons are presented in Supplement 1.

## 4 Discussion

The present study aimed to explore the positive and unwanted effects of a self-guided internet-based intervention (‘Restart’) for individuals with self-reported gambling problems. While previous research has demonstrated the effectiveness of self-guided internet-based interventions in reducing symptoms of gambling disorder (Rolvien et al., 2024; Sagoe et al., 2022), the current study contributes new insights into the potential of positive and unwanted effects of such interventions, an area that has received little attention in psychopathology research overall and has not yet been investigated in gambling studies.

### 4.1 Main findings

Overall, the findings indicated that PE of the intervention were reported more frequently than unwanted effects, which is consistent with a prior study investigating unintended effects in an internet-based interventions for body-focused repetitive behavior (BFRB; Baumeister et al., 2024). More than 60 % agreed on the item “After completing the online program, I gained confidence in my own abilities.”, suggesting an improvement in the experience of self-efficacy.

However, the occurrence of SE in 23.9 % of participants, MP in 38.6 %, and UP in 21.5 % raises important considerations for the implementation of such interventions. These rates appear to be higher compared to previous studies investigating significant symptom deterioration rates in internet-based interventions for depression (Ebert et al., 2016; Karyotaki et al., 2018) and across various disorders (mostly depression and anxiety disorders, Rozental et al., 2017), suggesting that the unique characteristics of gambling disorder might contribute to these effects. It may also be due to the different definitions/constructs that were assessed. In the aforementioned studies significant symptom deterioration was investigated, while in the present study various differentiated unintended effects were examined. A study that also used the PANEPS-I to assess positive and unwanted effects in an internet-based self-help intervention for BFRB found similar or even more unwanted effects (Baumeister et al., 2024). Also, the characteristics of the ‘Restart’ program may have influenced the observed outcomes. As a multi-component self-help intervention targeting both gambling behaviors and emotional/cognitive comorbidities, participants' engagement and responses may differ from those in interventions with a narrower focus, different intensity, or alternative delivery modes. Thus, both disorder- and intervention-specific factors should be considered when interpreting the findings.

The presence of SE, including deterioration of symptoms and feelings of stigmatization, aligns with earlier findings by Boettcher et al. (2014) and Fenski et al. (2021). These adverse effects could be attributed to the lack of personalized therapeutic support, which is a known limitation of self-guided interventions (Andersson and Titov, 2014). On the other hand, studies investigating why individuals with gambling problems avoid therapy suggest that many prefer to address their issues independently and avoid direct therapeutic contact, which would appear to contradict with therapeutic support in online interventions (Dąbrowska et al., 2017; Evans and Delfabbro, 2005). Consequently, the provision of such support should ideally be offered as an optional feature.

MP and UP, which were reported by a notable proportion of participants, may reflect challenges in delivering standardized content that resonates appropriately with diverse user needs and backgrounds (Linden, 2013). This is particularly relevant considering the complexity of gambling disorder, which often co-occurs with other mental health disorders (Grant and Chamberlain, 2020). Almost 20 % agreed with the item “The online program made me feel like I was to blame for my problems”, which on the one hand may indicate that the wording used in the online program could be indeed at least somewhat unfortunate. On the other hand, guilt and shame are very common feelings among people with gambling problems (Estévez et al., 2024), suggesting that such emotions may have been present regardless of program participation, especially as the program deliberately focused on a completely judgment-free approach. In addition, 13.3 % stated that the program put too much time and performance pressure on them, which could be addressed in future programs through individualization and adaptation to user needs. The likewise high level of agreement (12.1 %) for the item “I felt pressured by the online program to do things I didn't really want to do” could again be an important hint for us as developers to use less directive wording but could also be an indication of an ambivalent motivation for treatment. As with substance-related addictions, many individuals affected by gambling disorder – despite the serious consequences and a desire to abstain in the meantime – often still have a ‘desire’ to gamble again or they believe that nothing else would be as fun as to gamble.

PE significantly increased the likelihood that someone was a responder, in the sense that for each additional PE effect, the chance of being a responder increases by approximately 58.6 %. This suggests that PE may be an important mechanism for treatment success, in that they may represent a kind of indirect reward, potentially being experienced as a ‘substitute’ for the positive feelings associated with gambling. Unwanted effects (SE, MP and UP) had no significant impact on treatment success (*p* > .05), suggesting that unwanted effects are not hindering treatment success. It is conceivable that PE could act as a kind of reinforcer for the therapy. If patients experience PE such as improved well-being or more self-efficacy, this could strengthen their motivation to actively utilize the intervention. In addition, PE could contribute to patients developing healthier coping strategies. Unwanted effects, on the other hand, may only occur temporarily while the patient adapts to the intervention. In addition, it is normal during treatment for patients to be confronted with unpleasant feelings (e.g. guilt, shame). These effects may represent a necessary phase that does not diminish or affect the long-term effectiveness. It is also possible that the unwanted effects are not severe enough and therefore do not affect the success of the therapy sufficiently to show statistically significant effects.

Completers and non-completers differed only in gambling suspension status, with more non-completers reporting an active gambling suspension at baseline, suggesting that ongoing external restrictions may reduce adherence to self-guided interventions.

### 4.2 Strength and limitations

What we regard as a key strength of our study is the comprehensive assessment of both unintended positive and unwanted effects, which goes beyond merely identifying a deterioration of symptoms. We have adopted a more nuanced approach by considering the broader context in which these outcomes were generated. Specifically, we have differentiated between various unintended effects, such as positive side effects, traditional negative side effects, instances of malpractice, and unethical behavior. This holistic evaluation provides a more robust understanding of the consequences of the intervention and allows for a more informed interpretation of the findings.

Limitations of the study include its focus on self-reported gambling problems without clinical diagnoses, which may affect the generalizability of the results. Furthermore, the absence of a direct comparison group limits the ability to attribute unintended effects solely to the intervention. Also, the internal consistency of the UP subscale was low (α = 0.35), which limits the reliability of findings related to this subscale and suggests that results should be interpreted with caution. Additionally, negative effects were assessed only once at the six-week post-assessment, which limits causal interpretation and may be subject to recall bias. Future studies should include brief in-program assessments at multiple time points to better differentiate transient discomfort from enduring harm and to examine the temporal relation between negative effects and symptom change (Rozental et al., 2018). Given the modest sample size (*n* = 94, 46 responders), the regression analysis may have been underpowered to detect small effects, and non-significant findings should therefore be interpreted with caution. While the present model focused on the relationship between side-effect dimensions and treatment response, future work with larger samples could explore whether these effects remain when adjusting for baseline symptom severity or treatment engagement. Additionally, future research should include control groups and explore specific characteristics of participants who are more prone to experience unintended effects. Lastly, the study was not powered to examine interaction effects between program-related experiences and concurrent treatments, baseline severity, or demographic factors. Future research should test such moderation effects to clarify whether concurrent psychotherapy or medication use shape positive or unwanted outcomes in self-guided interventions.

## 5 Conclusion

In summary, while the ‘Restart’ program has benefits for individuals with gambling problems, a balanced approach that considers both unintended PE and unwanted effects is crucial. Overall, there were significantly fewer unwanted effects than PE. However, the results highlight the need for further research into strategies to minimize unwanted effects in self-guided internet-based interventions for gambling problems. Furthermore, future studies should examine larger samples that allow for more nuanced insights into the interaction of unintended effects with treatment. Improvements to the ‘Restart’ program, such as the integration of more personalized elements, adaptive content, measures to reduce experienced time and performance pressure as well as a more judgment-free language could potentially mitigate the risks of unwanted effects. In addition, the provision of optional therapeutic support or real-time assistance could reduce the likelihood of MP and UP. Promoting PE could be an important approach to improve the effectiveness of such interventions.

The following is the supplementary data related to this article.

Supplement 1 Exploratory comparison of completers versus non-completers regarding baseline characteristics and psychopathology (*N* = 119). Frequencies and means, and standard deviations and percentages in brackets. Supplement 1

## Declaration of competing interest

The authors declare the following financial interests/personal relationships which may be considered as potential competing interests: Steffen Moritz reports a relationship with Automaten Wirtschaftsverbände Info-GmbH that includes: funding grants. The Automaten-Wirtschaftsverbände Info GmbH has made financial contributions to the research unit of Dr Moritz outside the submitted work. No personal honoraria were received. No other disclosures were reported. If there are other authors, they declare that they have no known competing financial interests or personal relationships that could have appeared to influence the work reported in this paper.
