# Supplementary material for: Help or harm? Assessing positive and unwanted effects of a self-guided internet-based intervention for gambling problems
Source: Internet Interv. 2025 Dec 2;42:100893. doi: 10.1016/j.invent.2025.100893 (PMC12719061; doi:10.1016/j.invent.2025.100893)
Supplement: Supplement 1 — Exploratory comparison of completers versus non-completers regarding baseline characteristics and psychopathology (N = 119). Frequencies and means, and standard deviations and percentages in brackets. [file mmc1.docx]

Supplement 1. Exploratory comparison of completers versus non-completers regarding baseline characteristics and psychopathology (*N* = 119). Frequencies and means, and standard deviations and percentages in brackets.

|  | Completers  (*n* = 94) | Non-completers (*n* = 25) | Statistics |
| --- | --- | --- | --- |
| **Demographic characteristics** |  |  |  |
| Gender (% male) | 54 (57.4) | 19 (76.0) | χ²(1) = 2.87, *p* = .09 |
| Age in years | 34.70 (10.03) | 36.68 (13.65) | *t*(117) = .81, *p* = .21 |
| School leaving examination | 53 (56.4) | 7 (28.0) | χ²(6) = 11.24, *p* = .08 |
| Nationality (% German) | 84 (89.4) | 21 (84.0) | χ²(1) = .55, *p* = .46 |
| Professional status (% full-time employed) | 44 (46.8) | 14 (56.0) | χ²(6) = 2.15, *p* = .91 |
| **Treatment variables** |  |  |  |
| Psychotropic medication | 6 (6.4) | 1 (4.0) | χ²(1) = .20, *p* = .65 |
| Current usage of self-help | 5 (5.3) | 4 (16.0) | χ²(1) = 3.22, *p* = .07 |
| Currently in psychotherapy | 9 (9.6) | 3 (12.0) | χ²(1) = .13, *p* = .72 |
| Diagnosed gambling disorder | 4 (16.0) | 23 (24.5) | χ²(1) = .81, *p* = .37 |
| Currently in gambling suspension | 2 (1.7) | 7 (5.9) | χ²(2) = 23.25, *p* < .001 |
| **Psychopathology** |  |  |  |
| PG-YBOCS^a^ total score | 18.05 (7.94) | 17.88 (9.16) | *t*(117) = –.09, *p* = .46 |
| PG-YBOCS behavior | 8.75 (4.00) | 8.68 (4.90) | *t*(117) = –.07, *p* = .47 |
| PG-YBOCS thoughts | 9.31 (4.18) | 9.20 (4.61) | *t*(117) = –.11, *p* = .46 |

^a^Pathological Gambling Adaptation of Yale-Brown Obsessive-Compulsive Scale.
